# A Ship Detection Model Based on Dynamic Convolution and an Adaptive Fusion Network for Complex Maritime Conditions

**DOI:** 10.3390/s24030859

**Published:** 2024-01-28

**Authors:** Zhisheng Li, Zhihui Deng, Kun Hao, Xiaofang Zhao, Zhigang Jin

**Affiliations:** 1 School of Computer and Information Engineering, Tianjin Chengjian University, Tianjin 300384, China; lzs@tcu.edu.cn (Z.L.); dengzhihui0611@163.com (Z.D.); zhaoxftju@tju.edu.cn (X.Z.); 2 School of Electrical and Information Engineering, Tianjin University, Tianjin 300072, China; zgjin@tju.edu.cn

**Keywords:** adaptive ship detection, deep learning, YOLOv7, dynamic convolution, data augmentation

## Abstract

Ship detection is vital for maritime safety and vessel monitoring, but challenges like false and missed detections persist, particularly in complex backgrounds, multiple scales, and adverse weather conditions. This paper presents YOLO-Vessel, a ship detection model built upon YOLOv7, which incorporates several innovations to improve its performance. First, we devised a novel backbone network structure called Efficient Layer Aggregation Networks and Omni-Dimensional Dynamic Convolution (ELAN-ODConv). This architecture effectively addresses the complex background interference commonly encountered in maritime ship images, thereby improving the model’s feature extraction capabilities. Additionally, we introduce the space-to-depth structure in the head network, which can solve the problem of small ship targets in images that are difficult to detect. Furthermore, we introduced ASFFPredict, a predictive network structure addressing scale variation among ship types, bolstering multiscale ship target detection. Experimental results demonstrate YOLO-Vessel’s effectiveness, achieving a 78.3% mean average precision (mAP), surpassing YOLOv7 by 2.3% and Faster R-CNN by 11.6%. It maintains real-time detection at 8.0 ms/frame, meeting real-time ship detection needs. Evaluation in adverse weather conditions confirms YOLO-Vessel’s superiority in ship detection, offering a robust solution to maritime challenges and enhancing marine safety and vessel monitoring.

## 1. Introduction

Ship image detection technology is widely applied in various domains, such as maritime ship monitoring, shipping supervision, and maritime cruise search and rescue. However, in practical applications, different lighting conditions, complex backgrounds at sea, and stormy weather all increase the difficulty of ship detection [1]. This places higher demands on both accuracy and real-time ship detection. As computer vision technology rapidly advances, its applications are becoming increasingly widespread. These techniques have been gradually applied to ship detection and identification [2], which provides a new direction for maritime ship detection.

Ship image detection methods fall into traditional and deep learning methods. Traditional methods generally adopt support vector machine (SVM), histogram of oriented gradients (HOG), local binary pattern, and other algorithms for ship feature extraction and detection. For example, Feng et al. [3] present a multi-branch SVM approach to enhance the rapid detection of moving ships by incorporating effective multi-scale features. However, waves or changes in the background introduced higher computational costs to the model. Shi et al. [4] proposed an extended HOG method for detecting actual ships in candidate regions by computing a histogram of oriented gradients of local image regions. This method has the advantage of geometric invariance but increases the computational time, making it unsuitable for real-time applications. Zhu et al. [5] introduced a new texture operator to enhance feature extraction capabilities. However, in environments characterized by clouds, sea waves, and clutter, the method failed to extract detailed ship information, thereby reducing ship detection performance. Traditional methods exhibit limited portability and robustness across diverse scenes, and they are often susceptible to interference from complex backgrounds, noise, and low-light conditions [6]. Their real-time performance and accuracy are insufficient to meet task requirements. In the context of ship detection under adverse weather conditions, a series of methods for improving detection models have been proposed in references [7,8]. These methods are applied in complex maritime vessel monitoring systems under haze and low visibility conditions. The authors employ a direct detection technique. Another approach involves a two-step process: preprocessing the image to remove haze, and then conducting ship recognition. Song et al. [9] introduced a method for ship detection in hazy marine remote-sensing images. This method uses color polarization classification and haze concentration clustering to balance the remote sensing image (RSI) color and eliminate haze interference. The subsequent recognition of the processed image reduces the difficulty, but this method results in the loss of more image details. Liu et al. [10] proposed a novel image-dehazing algorithm based on color prior knowledge. This method achieves a higher accuracy in ship detection in thin cloud and mist environments, albeit with an increased computational burden due to the intricate preprocessing steps.

In contrast, deep learning possesses formidable feature learning capabilities, rendering it the prevailing approach in current ship detection technology. Object detection techniques in deep learning can be classified into two-stage and one-stage methods. For the two-stage detection method, Escorcia-Gutierrez et al. [11] introduced an enhanced Mask R-CNN model for improved recognition and classification of small ships in shipping. However, there remains a significant error in locating the ship’s contour edge region, indicating the necessity for further model accuracy improvement. Yu et al. [12] proposed an enhanced R-CNN method called Ship R-CNN, which improves ship detection accuracy in scenarios with complex backgrounds and minimal differentiation between ships and distant shores. However, this method did not account for ship recognition under nighttime conditions. Li et al. [13] improved the Cascade R-CNN method for more accurate small-ship detection. However, it faces efficiency challenges in recognizing redundant features. In the one-stage detection methods, the most representative algorithm is the You Only Look Once (YOLO) family. The algorithms employ a direct regression approach to make predictions over the entire image, effectively improving detection speed while maintaining accuracy, and are thus widely used in ship detection. Specifically, Guo et al. [14] presented LVENet, an enhancement network for improved low-light maritime vessel detection by enhancing image channel luminance. However, the network does not account for the challenges of rainy and foggy weather conditions, and its model exhibits limited generalization. Guo et al. [15] improved the deblurring and defogging performance of the model by enhancing the fused image feature information, but their method is limited by the dark light environment and noise interference, which increases the uncertainty of the prediction results.

In summary, although some research results have been achieved in ship image detection under complex sea conditions, challenges persist. First, the presence of various ship types with significant size variations between classes and small target scales poses difficulties for target detection. Secondly, adverse factors like sea haze, uneven illumination, and low visibility in complex backgrounds can degrade imaging quality. Extracting effectively ship features from the ocean background remains a challenge for algorithms. This paper presents a ship detection model tailored for complex sea state images, with three key contributions.

1. We propose an improved real-time ship detection model based on YOLOv7 (YOLO-Vessel), specifically designed to address ship detection challenges in the complex sea conditions mentioned above.
2. A backbone network called Efficient Layer Aggregation Networks and Omni-Dimensional Dynamic Convolution (ELAN-ODConv) with strong feature extraction capability is designed to reduce false and missed detections. Then, a network termed Efficient Layer Aggregation Networks Head and Space-to-depth and Convolution (ELANH-SPDC) is introduced at the head to achieve fine-grained detection and identification of ships. In addition, a new prediction network structure named ASFFPredict is designed, which adaptively learns each feature layer’s weights and can fuse each scale’s feature information more efficiently.
3. To adapt ship detection under different adverse weather conditions, this paper proposes a ship dataset under adverse weather conditions, which is then artificially synthesized using physical haze, rain, snow, and low light algorithms, and experiments are conducted in real scenarios to verify the detection accuracy and operation efficiency of this model.

In this paper, Section 2 introduces some related research work. Section 3 describes the presented ship detection model. Section 4 analyzes the experimental performance of the YOLO-Vessel model and showcases ship detection results in real environments. Section 5 concludes this article.

## 2. Related Work

The YOLO series of models has garnered extensive attention in recent years, and researchers have achieved a series of advancements in ship detection research based on the YOLO framework. For example, Yao et al. [16] employed the YOLOv8 model for multi-class ship detection, improving ship recognition accuracy. However, their dataset was not comprehensive enough and lacked training data for large-sized vessels. Furthermore, Zhao et al. [17] proposed a detection model named YOLOv7-sea, incorporating attention mechanisms to enhance focus on regions containing vessels of interest. However, this approach fails to extract multi-scale ship features and may lead to erroneous detections. To tackle inadequate feature extraction, dynamic convolutions have gradually found applications across various domains in deep learning. The representative omni-dimensional dynamic convolution (ODConv) [18] is a novel convolutional operation capable of dynamically adjusting convolution kernels to effectively capture multi-dimensional features in data, thereby enhancing the model’s performance in detection tasks. Cheng et al. [19] integrated dynamic convolution modules into shallow networks, enhancing the model’s efficiency in ship recognition under complex backgrounds. However, this approach encounters issues such as false positives for small-sized vessels and prolonged model training times. Complex maritime ship target recognition often results in false positives, and due to intricate backgrounds and noise interference, it may also fail to recognize small-sized vessels. Chen et al. [20] presented a multi-scale ship detection model for complex scenes. They incorporated the ASPP module to expand the receptive field while reducing feature loss for small-sized vessels. However, this model did not take into account the time cost.

To further improve the detection of small ships in complex sea conditions, SPDC has unique advantages in detecting small targets and low-resolution images. The SPDC structure consists of space-to-depth and convolution [21], where space-to-depth is a transformation layer that downsamples the feature maps in the CNN using image transformation techniques while retaining all the channel information to enhance small-size feature extraction. Ma and Pang et al. [22] presented an SP-YOLOv8s detection model that enhances the fine-grained feature information during downsampling, improving the accuracy of detecting small objects. However, this gain in accuracy comes at the cost of increased computational complexity. Multi-scale fusion networks have found widespread application in deep learning models. Zhang et al. [23] presented an improved model built upon YOLOv7-tiny. This model integrates multi-scale residual modules, enhancing ship detection performance in complex water surface environments. However, its performance may degrade when detecting target vessels at more minor scales.

To enhance multi-scale feature information, the adaptive spatial feature fusion (ASFF) mechanism dynamically tunes the weights assigned to feature maps. This dynamic adjustment empowers the model to get information at varying scales and hierarchical levels [24], resulting in a more comprehensive feature fusion [25]. Guo et al. [26] proposed a lightweight LMSD-YOLO model to create a real-time maritime vessel detection model with a reduced parameter count. The model achieves an adaptive fusion of multi-scale features. However, its feature extraction capability falls short in low-visibility images and noise interference, leading to potential false negatives for small vessels.

The current YOLO algorithm has been developed to version 8 (YOLOv8). Compared to the previous version, YOLOv7, YOLOv8 introduces the convolution to fusion structure, reducing the number of convolution modules, resulting in faster detection speeds. However, this speed enhancement comes at the cost of sacrificing some detection accuracy. Consequently, YOLOv8 might exhibit reduced ship detection accuracy in complex environments. YOLOv7 incorporates the efficient layer aggregation networks (ELAN) structure to facilitate multi-branch gradient flow feature extraction [27]. This design enhances the model’s detection performance, making it better suited for ship detection in complex maritime conditions. The YOLOv7 consists primarily of four parts: input, backbone network, head network, and prediction network. The ELAN [28] combines VoVNet and CSPNet [29]. It enables the deep network to converge more effectively without changing the original model structure gradient propagation path and continuously enhances its learning capability. The head network enhances its feature fusion capability with the SPPCSPC module and path aggregation network (PANet).

Given the challenges related to work poses and the difficulty of detecting small vessels in complex environments, further improvements are needed for the YOLOv7 model. Dynamic convolutions offer advantages in capturing multi-dimensional features, and SPDC is adept at enhancing small object detection at low resolutions. Both of these techniques contribute to improving feature information extraction. ASFF can effectively merge multi-scale features to enrich feature detection information. Therefore, this study introduces dynamic convolutions, SPDC, and ASFF networks to the basic YOLOv7 model to enhance detection outcomes.

## 3. Proposed Detection Framework

**Figure 1:**
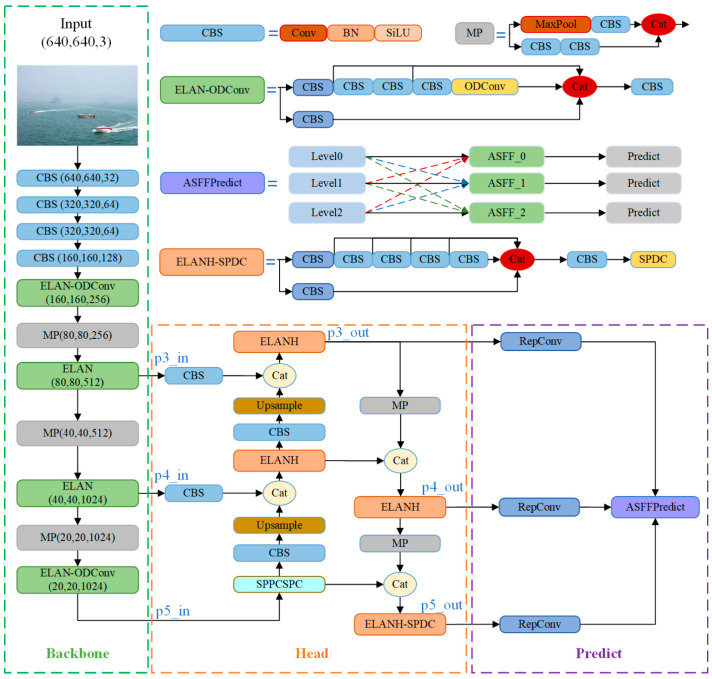
Entire structure of the YOLO-Vessel.

One needs to strengthen the network’s feature extraction capabilities and optimize information flow to enhance the model’s effectiveness in detecting ships under challenging sea conditions. Therefore, this paper optimizes the backbone, head, and prediction network components of YOLOv7 by leveraging the advantages of ODConv, SPDC, and ASFF. As illustrated in Figure 1, the YOLO-Vessel model comprises three main components: the ELAN-ODConv backbone network structure, ELANH-SPDC head structure, and ASFFPredict prediction network structure.

### 3.1. Backbone Network

**Figure 2:**
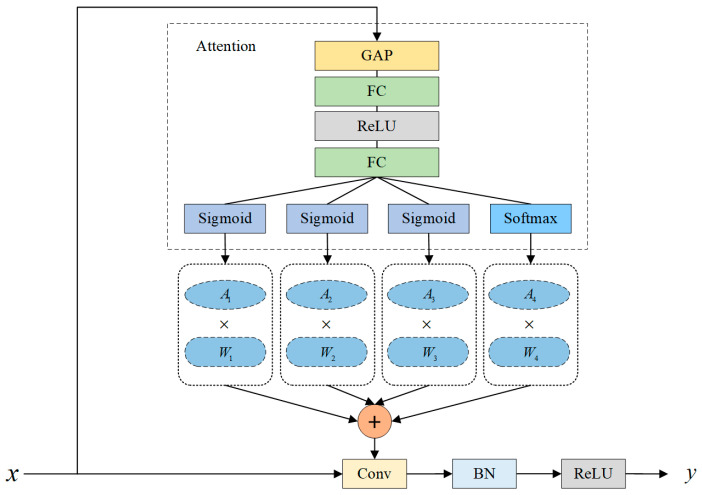
Structure of ODConv.

The YOLO-Vessel backbone network incorporates CBS, ELAN-ODConv, and MP modules. The CBS module comprises a convolution layer, with the SiLU activation function applied afterward, followed by the addition of a batch normalization (BN) layer. The ELAN-ODConv module includes the CBS module and the ODConv unit. This design enhances the network’s learning capabilities by extending and merging bases while preserving the original gradient path. In each ELAN-ODConv structure, downsampling is achieved by compressing the feature map scale using a 3 × 3 convolution kernel with a stride of 1 and zero padding. Then, the feature map passes through two branches: one enters the CBS module, and the other enters multiple CBS modules and an ODConv structure. Finally, the outputs of the two branches are operated with Concat and partially transformed using a 1 × 1 convolution module to improve the learnability of the model. As shown in Figure 2, the design idea of ODConv is to generate a new feature map by performing element-wise multiplication and addition of four convolution kernels, each of the same size and dimension, while considering their corresponding attention weights, and finally, by using a convolution operation.

ODConv is a more generalized form of dynamic convolution, with its computational form as follows:

(1) $$y=\left(A_{1}\times W_{1}+A_{2}\times W_{2}+A_{3}\times W_{3}+A_{4}\times W_{4}\right)\times x$$

$A_{1}$, $A_{2}$, and $A_{3}$ are three newly introduced attention weights, representing the weights associated with the spatial position of the convolution kernel, input channels, and output channels, respectively. $A_{4}$ is the attention weight corresponding to the number of convolutional kernels.

Figure 2 shows that ODConv learns the four attention weights of the convolutional kernel in parallel along four dimensions. The input feature map x is first subjected to a global average pooling (GAP) operation and then passed through a Fully Connected (FC) Layer—ReLU Activation Layer—Fully Connected (FC) Layer structure. Finally, a set of attention weights $\left\{A_{1},A_{2},A_{3},A_{4}\right\}$ is obtained at the output of the sigmoid and softmax activation function layers. Specifically, $A_{1}$, $A_{2}$, and $A_{3}$ are generated by the Sigmoid activation function, i.e.,

(2) $$Sigmoid(x)=\frac{1}{1+e^{-x}}$$

This function maps input values to the interval (0,1). $A_{4}$ is generated by the Softmax activation function, i.e.,

(3) $$Softmax(x_{i})=\frac{e^{x_{i}}}{\sum_{j=1}^{n}e^{x_{j}}}$$

Here, x represents the *i*th element of the input vector. Softmax produces a set of probability values, introducing normalized constraints to simplify the learning of $A_{4}$. The final weighted values of each group of convolutional kernels are used to generate output features. Compared with ordinary dynamic convolution, which only considers the single factor of the number of convolution kernels, ODConv adds multiple-dimensional information so that the input features can obtain rich contextual information.

**Figure 3:**
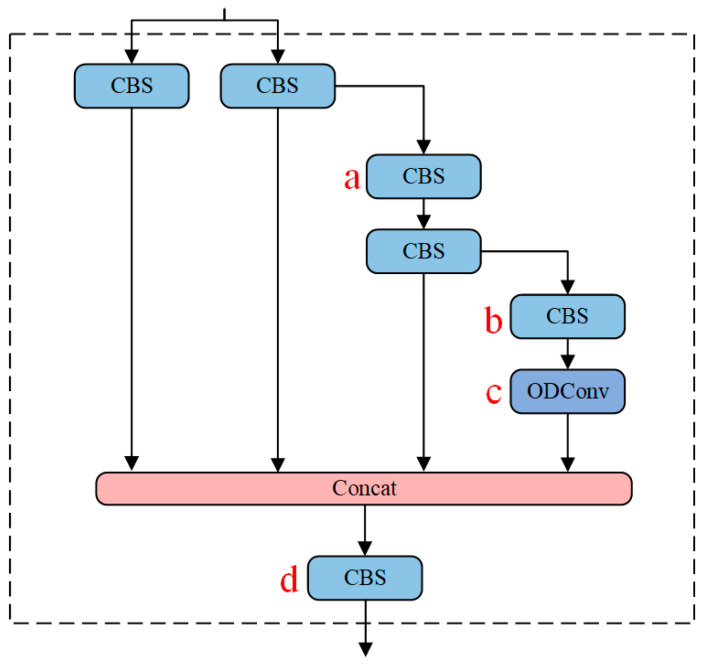
Structure of ELAN-ODConv. The ODConv is introduced into the ELAN module, where positions a, b, c, and d are highlighted in red, representing the locations where ordinary convolutions are replaced with ODConv.

In this study, ODConv is employed to achieve the goal of a more precise model without increasing the network’s width and depth. Figure 3 presents four variations of ELAN-ODConv types, namely ELAN-ODConv-a, ELAN-ODConv-b, ELAN-ODConv-c, and ELAN-ODConv-d, designed with ODConv at different positions in the backbone network of the YOLOv7-Vessel. Experimental results (as detailed in Section 4.5.2) indicate that replacing the ordinary convolution module with ODConv at position c within the ELAN module results in the maximum mAP value. Therefore, ELAN-ODConv-c is selected as the preferred ELAN-ODConv configuration.

### 3.2. Head Network

YOLO-Vessel’s head network consists of the PANet structure, the SPPCSPC module, and the ELANH-SPDC module. The feature information extracted from the backbone network is generated in the p3_in, p4_in, and p5_in layers at the three scales of 80 × 80, 40 × 40, and 20 × 20, respectively, and output to the head network. Subsequently, the 20 × 20 scale feature map is first upsampled using the SPPCPSC module to perform a Concat operation with the 40 × 40 scale feature map. Then, the output features are upsampled to perform a second fusion with the 80 × 80 scale feature map to further the fusion between adjacent scales of the feature map. However, in the multiscale structure, as the depth of the network grows, the location information of the feature map is weakened, and the resolution of the map gradually decreases, which leads to the degradation of the model’s detection performance. Accordingly, we introduce an ELANH-SPDC module, incorporating the SPDC module into the head network’s tail. This enhancement aims to sharpen the model’s attention towards low-resolution and small objects, particularly in remote sea areas, while also boosting recognition performance for low-resolution feature maps. 

**Figure 4:**
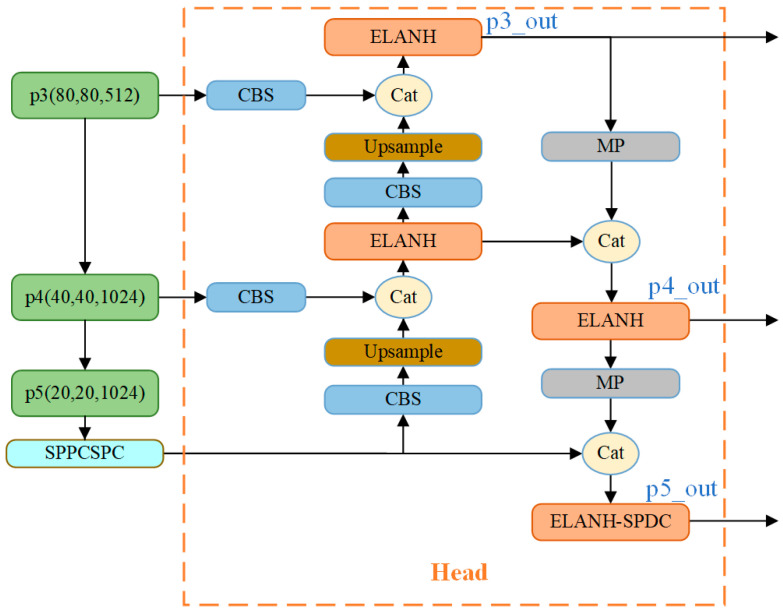
The head network is equipped with the ELANH-SPDC module.

As shown in Figure 4, this study incorporates the SPDC module into three head network positions: p3_out, p4_out, and p5_out. When the feature maps enter the Head, the channel dimension reaches its maximum while the network resolution reaches its minimum. Introducing features learned by SPDC at this stage is crucial for detecting low-resolution features. Experimental results (as detailed in Section 4.5.1) indicate that inserting SPDC at position p5_out in the head network yields the optimal detection performance for the model. Therefore, the SPDC designed at position p5_out is employed to create the ELANH-SPDC module, which is then integrated into the overall head network structure.

The ELANH-SPDC module is the main feature extraction module located at the output position of p5_out in the head network, which divides the gradient stream into network paths of different lengths. The Concat operation fuses the features of each branch, ultimately replacing the original 1 × 1 standard convolution with the SPDC module to obtain more effective feature information. This approach preserves more fused, detailed features and optimizes the model’s detection accuracy. We set X as the original feature map, $X_{1}$ as the intermediate feature map, $X_{2}$ as the final feature map, f as the subfeature map, scale as the feature map scale scaling factor, S as the feature map length and width dimension values, $C_{1}$ as the feature map depth value, and $C_{2}$ as the convolution kernel depth value. Space-to-depth cuts a feature map X of size $S\times S\times C_{1}$ into a series of subfeature maps, and each subfeature map $f_{x,y}$ is formed by all entries $X\left(i,j\right)$ of i+x and j+y divided by scale. The SPDC calculation equation is as follows:

(4) $$\begin{array}{c} f_{0,0}=X\left[0:S:scale,0:S:scale\right],\\ f_{1,0}=X\left[1:S:scale,0:S:scale\right],\\ \ldots \\ f_{scale-1,0}=X\left[scale-1:S:scale,0:S:scale\right];\\ \vdots \\ f_{0,scale-1}=X\left[0:S:scale,scale-1:S:scale\right],\\ f_{1,scale-1}=X\left[1:S:scale,scale-1:S:scale\right],\\ \cdots \\ f_{scale-1,scale-1}=X\left[scale-1:S:scale,scale-1:S:scale\right] \end{array}$$

**Figure 5:**
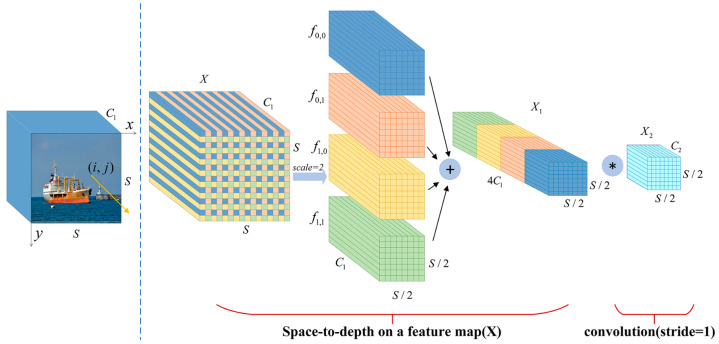
Structure of SPDC. The symbol “*” represents the convolution operation.

The graphical process is given in Figure 5, where four subfeature maps $f_{0,0}$, $f_{0,1}$, $f_{1,0}$, $f_{1,1}$ are obtained when scale=2, each with size $\left(S/2,S/2,C_{1}\right)$, which is equivalent to twice the downsampling of the original feature map X. Then, all the subfeature maps are connected along the channel dimension to obtain the intermediate feature map $X_{1}$, where the $X_{1}$ spatial dimension is reduced by a scale factor, and the channel dimension is increased by a scale factor. The space-to-depth layer converts the original feature map $X\left(S,S,C_{1}\right)$ into an intermediate feature map $X_{1}\left(S/scale,S/scale,{scale}^{2}C_{1}\right)$ with feature discrimination information. A convolutional layer with a $C_{2}$ filter is added in Figure 5 to achieve further transformation from the intermediate feature map $X_{1}$ to the final feature map $X_{2}$. The step size of this convolutional layer is set to 1 to retain the maximum amount of discriminative feature information. Therefore, we introduce the SPDC structure into the ELANH structure of the head network, which can effectively improve the detection performance of the model for low-resolution and small ships at sea.

### 3.3. Prediction Network

**Figure 6:**
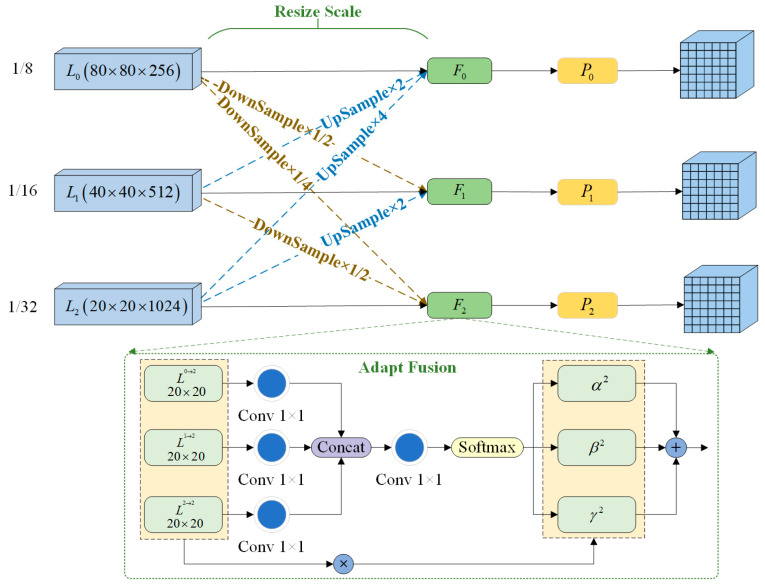
The process of adaptively spatial feature fusion.

The YOLO-Vessel model uses a multiscale prediction method in the prediction network part, where the input 640 × 640 scale images are downsampled in the backbone structure by factors of 8×, 16×, and 32×. Then, the output of the head network yields 80 × 80 scale feature maps p3_out, 40 × 40 scale feature maps p4_out, and 20 × 20 scale feature maps p5_out. Among them, p3_out is used for small-scale target detection, p4_out is used for medium-scale target detection, and p5_out is used for large-scale target detection. As depicted in Figure 6, this paper introduces an adaptively spatial feature fusion network structure named ASFFPredict. Utilizing ASFF, the network learns optimal fusion weights to accentuate feature layers that contain more information about small targets. Subsequently, features from each feature layer are fused to ensure that elements with higher weights dominate the expression in the resulting feature map. The introduction of ASFF between the head network and YOLO head aims to enhance the model’s detection performance for small maritime targets, providing an innovative solution for ship detection problems in maritime regions.

We select three layers of feature map output from the head network for fusion. In Figure 6, $L_{0}\left(L_{0}=80\times 80\times 256\right)$, $L_{1}\left(L_{1}=40\times 40\times 512\right)$, and $L_{2}\left(L_{2}=20\times 20\times 1024\right)$ denote the feature maps involved in adaptive fusion. Direct feature fusion cannot be performed since these three feature maps have different scales and channels. Therefore, the resolution and channels of each feature layer need to first be adjusted to be the same. Taking $L_{2}$ layer fusion as an example, the fused feature map $L_{2}$ is labeled $P_{2}$, and then, the three spatial weights from $L_{0}$, $L_{1}$, and $L_{2}$ to $P_{2}$ are labeled $\alpha^{2}$, $\beta^{2}$, and $\gamma^{2}$, respectively. The expressions are as follows:

(5) $$P_{ij}^{2}=\alpha_{ij}^{2}\cdot L_{ij}^{0\to 2}+\beta_{ij}^{2}\cdot L_{ij}^{1\to 2}+\gamma_{ij}^{2}\cdot L_{ij}^{2\to 2}$$

where $\alpha_{ij}^{2}+\beta_{ij}^{2}+\gamma_{ij}^{2}=1$, $\alpha_{ij}^{2}{,\beta }_{ij}^{2}{,\gamma }_{ij}^{2}\in \left[0,1\right]$, and $\alpha_{ij}^{2}$, $\beta_{ij}^{2}$, and $\gamma_{ij}^{2}$ are normalized scalars that are calculated using the Softmax function. The expressions are as follows:

(6) $$\alpha_{ij}^{2}=\frac{e^{\lambda_{\alpha_{ij}}^{2}}}{e^{\lambda_{\alpha_{ij}}^{2}}+e^{\lambda_{\beta_{ij}}^{2}}+e^{\lambda_{\gamma_{ij}}^{2}}}$$

$L_{ij}^{0\to 2}$ and $L_{ij}^{1\to 2}$ denote the feature maps with transformed scales, which are transformed from the feature maps of the $L_{0}$ layer to the $F_{2}$ layer and from the feature maps of the $L_{1}$ layer to the $F_{2}$ layer. In this process, a 3 × 3 convolution is first used to downsample the $L_{0}$ layer four times and the $L_{1}$ layer two times, thus adjusting the feature maps of all layers to a 20 × 20 scale size. Then, the other feature layers are fused and upsampled using nearest neighbor interpolation. Next, the feature channels of $L_{ij}^{0\to 2}$ and $L_{ij}^{1\to 2}$ are adjusted to 1024 using 1 × 1 convolution, and the feature map scale is kept constant. Finally, $L_{ij}^{2\to 2}$ and the transformed $L_{ij}^{0\to 2}$ and $L_{ij}^{1\to 2}$ are subjected to the Concat operation, and each of the three feature maps are multiplied by their respective weights; the results are then summed to get the feature map $P_{2}$. The weight parameters are learned from the convolutional layer output using gradient back-propagation, and the weights can be adaptively adjusted in the feature fusion process. The adaptively spatial feature fusion process achieves a better fusion of features at different scales, effectively recognizes small and multiscale objects, and enhances the capability for maritime vessel detection.

## 4. Experiments

### 4.1. Dataset Preparation and Data Augmentation

The datasets we use are derived from two public datasets and one handcrafted dataset, including the public ship dataset SeaShips7000 [30] and the ship dataset provided by the 2nd International Challenge for Intelligent Perception of Marine Targets in 2021, with 200 and 4300 images, respectively. The mixed public dataset covers six ship categories: bulk carrier, sailboat, container ship, yacht, cruises, and fishing boat. This dataset is divided into an 8:1:1 ratio for training, validation, and testing. There are 415 pictures in the homemade dataset collected using a fixed shooting device in the Yangtze River waters of the Chongqing section. The shooting device used a Hikvision 23× zoom monitoring dome with a resolution of 2560 pixels (horizontal) × 1440 (vertical) pixels and shot ship images containing three real scenes of normal, rain, haze, and dawn from different positions and angles. Due to the lack of snowy weather, the ship images in snowy weather come from real snowy ship pictures collected by web crawlers. The homemade dataset includes ship pictures captured in various weather conditions: normal clear, rain, haze, snow, and dawn. Each weather type constitutes 20% of the dataset, allowing the model’s performance in detecting ships under real-world environmental conditions to be evaluated.

**Figure 7:**
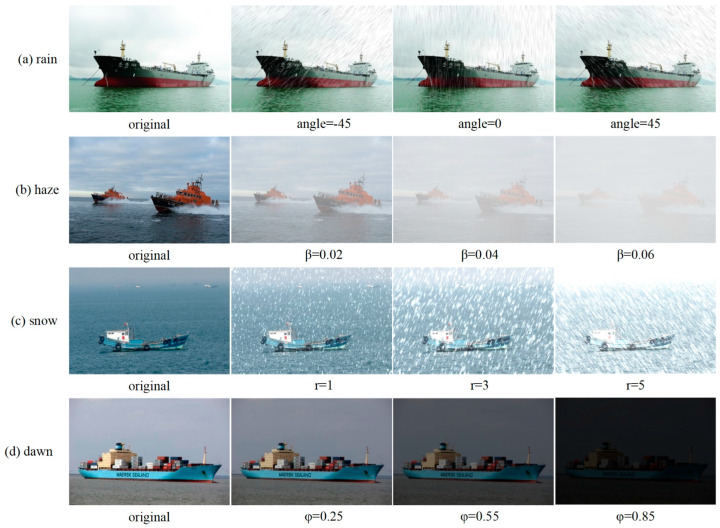
Synthesized ship images in adverse maritime weather conditions. (**a**) Rainy weather images, including the original and rainy images with rain streak angles of −45 degrees, 0 degrees, and 45 degrees. (**b**) Haze images, including the original image and images with haze concentrations of 0.02, 0.04, and 0.06. (**c**) Snowy weather images, including the original and snowy images with 1, 3, and 5 snow levels. (**d**) Dawn images, including the original and dawn images, have low light coefficients of 0.25, 0.55, and 0.85.

Usually, images acquired in complex adverse weather are visually richer and better match the actual complex sea-going ship conditions. However, acquiring large quantities of realistic ship images in adverse weather conditions in real sea environments is challenging, which makes it necessary to use synthetic images. In this paper, the above-mixed public dataset is artificially synthesized with images of ships under severe weather conditions [31], and the synthesized dataset is named “SeaShips_weather.” It is a synthesis of rain, haze, snow, and dawn weather images based on the RGB layer stacking algorithm, atmospheric scattering model, and retinex theory, as shown in Figure 7.

1. Rain patterns with different tilt angles of −45, 0, and 45 degrees are randomly added to the preprocessed image to synthesize the rain ship image. The expressions of synthetic rain are as follows:
  (7) $$A\left(x,y\right)=N\left(x,y\right)+M\left(x,y\right)+\delta$$
  A(x,y) denotes the synthesized rain image, $\left(x,y\right)$ denotes any pixel in the image, N(x,y) denotes the original image, M(x,y) denotes the rain pattern layer, and δ denotes a random luminance value, as shown in Figure 7a.
2. The haze can significantly degrade image quality during detection. To simulate ship scenes in such conditions, we employ an atmospheric scattering model to synthesize hazy sky images. The formula for creating artificial haze is as follows:
  (8) $$I\left(x,\lambda \right)=g\left(\lambda ,x\right)R\left(x,\lambda \right)+L_{\infty }\left(1-g\left(\lambda ,x\right)\right)$$
  $I\left(x,\lambda \right)$ is the synthesized dense haze image, $R\left(x,\lambda \right)$ is the original image, the parameter x is any pixel in the image, λ is the wavelength of light, $L_{\infty }$ is the value of scattered atmospheric light at infinity, and $g\left(\lambda ,x\right)=e^{-\beta \left(\lambda \right)d\left(x\right)}$ is the light propagation function, in which β is the atmospheric scattering factor and $d\left(x\right)$ is the distance of the target object. Adjusting the atmospheric scattering factor $\beta \in \left\{0.02,0.04,0.06\right\}$ synthesizes the sea haze images of different degrees, as shown in Figure 7b.
3. As depicted in Figure 7c, the snowflake texture is randomly added to the original image by adjusting the snow amount value $r\in \left\{1,3,5\right\}$ to synthesize a ship image on a snowy day. The expression of the synthesized snowflake is as follows:
  (9) $$B\left(x,y\right)=E\left(x,y\right)+R\left(x,y,r\right)+\beta$$
  $B\left(x,y\right)$ denotes the synthesized snow day image, $\left(x,y\right)$ denotes any pixel in the image, $E\left(x,y\right)$ denotes the original image, $R\left(x,y\right)$ denotes the rain pattern layer, and β denotes the random luminance value in the image.
4. Dawn weather tends to cause low brightness, low contrast, and detail loss in the image. The ship image under dawn weather is synthesized using the retinex algorithm, as shown in Figure 7d, where the attenuation coefficient $\varphi \in \left\{0.25,0.55,0.85\right\}$ changes the image brightness value. The expression of the synthesized dawn image is as follows:
  (10) $$P\left(x,y\right)=Q\left(x,y\right)\cdot L\left(\varphi \right)$$
  $P\left(x,y\right)$ is the synthesized dawn image, $Q\left(x,y\right)$ is the original image, and $L\left(\varphi \right)$ is the spatially smoothed luminance function.

### 4.2. Experimental Environment 

The experiments were conducted with the following configurations: an Ubuntu 18.04.6 operating system, a Tesla V100 GPU with 32GB of memory (Austin, TX, USA), and an Intel(R) Xeon(R) Platinum 8163 CPU (Santa Clara, CA, USA). To accelerate the computations, we employed CUDA 10.2 and cuDNN 7.6.5. The training of our proposed model and the comparison models was carried out using the PyTorch 1.7.1 framework.

### 4.3. Experimental Setup

The crucial details of the key parameters for training the network model in this study are as follows: the input image size is set to 640 × 640, the initial learning rate is 0.001, momentum is 0.937, weight decay is 0.0005, the batch size is 4, each training epoch duration is 62 s, Mosaic is set to 1.0, and the optimizer employs stochastic gradient descent (SGD) with cosine learning rate decay strategy. Other parameters adopt default values from YOLOv7. Mosaic data augmentation enriched the training dataset by introducing four images simultaneously, which underwent flipping, zooming, and splicing operations to diversify the detection scenarios. The training process comprised 150 epochs, totaling 135,000 iterations.

### 4.4. Evaluation Index

To quantitatively evaluate the detection effectiveness of the proposed model, seven evaluation metrics are introduced to examine the performance, including precision (*P*), recall (*R*), average precision (*AP*), mean average precision (*mAP*), giga floating point operations per second (GFLOPS), inference time (Infer), and F-Measure (*F*1). Their calculation formulae are shown below.

(11) $$Precision=\frac{N_{TP}}{N_{TP}+N_{FP}}$$

(12) $$Recall=\frac{N_{TP}}{N_{TP}+N_{FN}}$$

(13) $$AP=\int_{0}^{1}P\left(r\right)dr$$

(14) $$mAP=\frac{\sum_{i=1}^{m}AP_{i}}{m}$$

(15) $$F1=2\cdot \frac{Precision\cdot Recall}{Precision+Recall}$$

where $P\left(r\right)$ denotes the P-R curve, m denotes the number of detected ship categories, $N_{TP}$ denotes the number of correctly detected ships, $N_{FP}$ denotes the number of mis-detected ships, and $N_{FN}$ denotes the number of undetected ships.

### 4.5. Experimental Results and Analysis

#### 4.5.1. Impact of SPDC Integration on Network Performance

Incorporating the SPDC module into the YOLOv7 model enables effective learning of ship features under complex weather conditions, which is particularly crucial for detecting low-resolution images. A meticulous evaluation and pre-selection of three potential scenarios was conducted to determine the optimal location for introducing SPDC into the network. The objective was to identify the optimal scenario that achieves the highest performance regarding mAP and F1 metrics while minimizing the computational complexity of the model’s GFLOPS. The first scenario involves introducing SPDC at the p3_out output position of the head network, the second scenario selects the p4_out output position, and the third scenario opts for the p5_out output position. As shown in Table 1, through a comparative analysis of these scenarios, the performance is optimal when SPDC is positioned at p5_out. Despite a slight increase in inference time, the model achieves an mAP of 77.6%, F1 of 76, and a significant reduction in model complexity, with GFLOPS reaching 94.8.

#### 4.5.2. Results of Replacing Standard Convolution with ODConv

This section analyzes the performance of applying ODConv at different positions in the ELAN module of YOLOv7. The ELAN layer in YOLOv7 consists of seven positions for ordinary convolutions. The third, fifth, sixth, and seventh positions are selected as potential locations for replacing ordinary convolutions with ODConv. As illustrated in Figure 3, four positions in the ELAN module are labeled as a, b, c, and d, representing four improved modules. The comparative detection results are presented in Table 2.

The experimental results indicate that the YOLOv7-ODConv-c model achieves better performance at position c. Compared to the YOLOv7 model, the YOLOv7-ODConv-c model shows a slight increase in inference time due to the increased learning weight dimensions of ODConv. However, it achieves an improvement of 1.6% in mAP, a 2% increase in F1, and a reduction in GFLOPS to 100.9. In summary, with a marginal sacrifice in speed, the YOLOv7-ODConv-c model significantly enhances ship detection accuracy.

#### 4.5.3. Ablation Experiment

This section verifies the effectiveness of ELAN-ODConv, ELANH-SPDC, and ASFFPredict for ship detection tasks in adverse weather conditions at sea. Based on the YOLOv7 model, three different network models were constructed sequentially, introducing new modules combined with varying network structures, as shown in Table 3. Here, √ indicates the incorporation of the corresponding improvement module, while × suggests the absence of the respective improvement module.

YOLO-ES introduces a single new module, YOLO-OS introduces two new modules, and YOLO-Vessel incorporates all three new modules.

**Figure 8:**
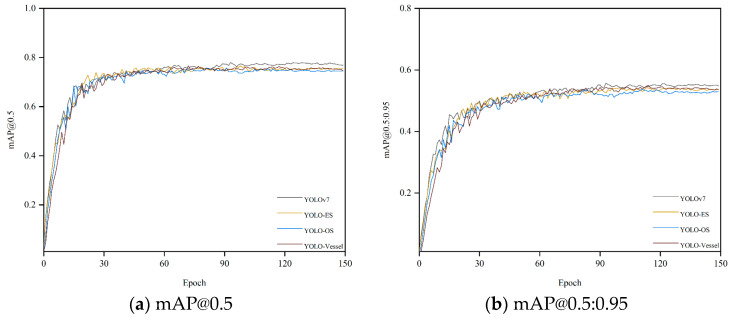

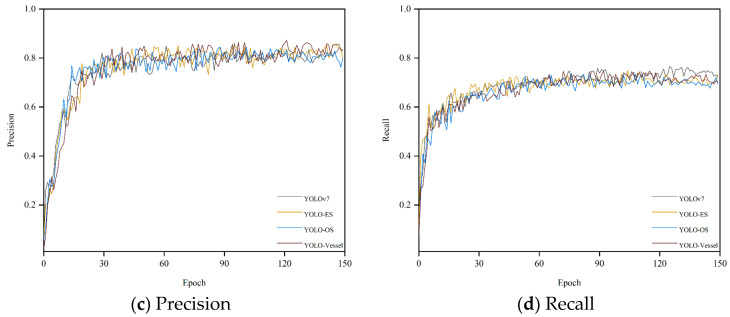
Performance comparison of training different improved models on the synthetic weather ship dataset.

We conducted several experiments on a synthetic weather ship dataset. Figure 8 shows the mAP, precision, and recall curves for all detectors during model training. All curves rise gently and converge quickly, indicating that the model is well-trained and not overfitted. As depicted in Figure 8a,b, the overall training curves exhibit minimal fluctuations. However, Figure 8c,d reveal more significant volatility during the ascending phase of the overall training curve, although these fluctuations minimally impact detection accuracy. Due to the introduction of ODConv and SPDC, the model’s detection capability has been enhanced. YOLO-Vessel improves precision from 82.8% to 83.7% compared to the original YOLOv7 model. Additionally, after training for 150 epochs, the recall, mAP@0.5, and mAP@0.5:0.95 indices reach 70.2%, 74.5%, and 53.6%, respectively. The model performs well in detecting ship images.

**Figure 9:**
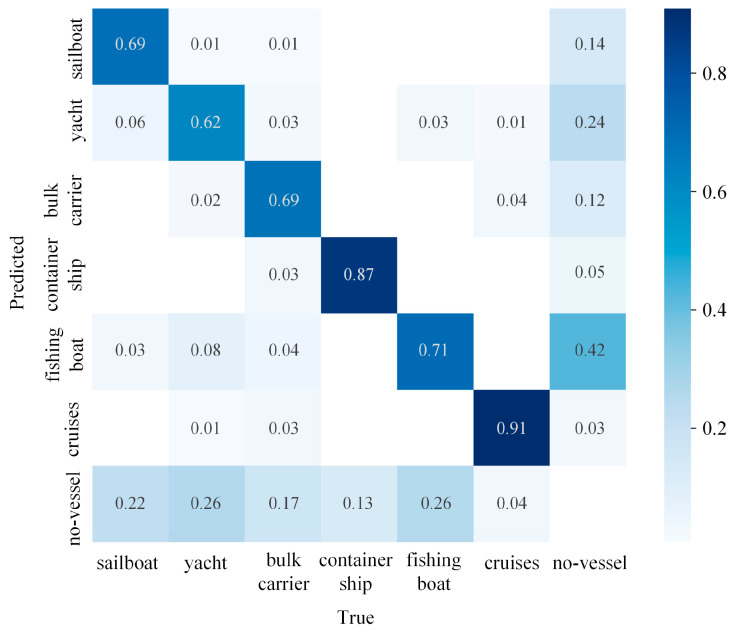
Confusion matrix for the YOLO-Vessel model.

During the individual testing process for each image, predictions and actual values for each category were recorded, resulting in a confusion matrix, as shown in Figure 9. It can be observed that there are misclassifications between cargo ships and cruise ships, primarily due to the similar features of these two types of vessels and the blurriness of ships in adverse maritime weather conditions, making them easily confused. To address this, a “no-vessel” category has been added to the detection, representing the absence of any vessel. Fishing boats also experience misclassifications, mainly due to their small size. In complex maritime weather conditions, interference from background noise can easily result in misidentifying fishing boat classes as “no-vessel” classes. Therefore, adverse weather conditions significantly impact the detection of ships.

Next, the results of different improved models of YOLOv7 were compared, as shown in Table 4.

The following conclusions were drawn from Table 4:

1. YOLO-Vessel is the best performer in the YOLO series regarding mAP. Compared with the original YOLOv7, its mAP performance improved by 2.3%. It can be observed that the original YOLOv7 has the lowest mAP value and unsatisfactory detection results. Compared with the original YOLOv7 model, all three improved network models have improved mAP. Among them, the overall mAP values for ship detection increased by 1.6%, 1.7%, and 2.3%, respectively. The analysis shows that YOLO-ES demonstrates higher accuracy in recognizing cruise ships. Additionally, the improved models offer better recognition performance for small to medium-sized sailboats, yachts, and fishing boats. Compared to YOLOv7, YOLO-ES exhibits improved recognition of fishing boats, with an increase in AP value by 0.9%, indicating that the ELANH-SPDC structure can enhance the feature information for low-resolution targets during feature fusion, demonstrating exemplary performance in detecting low-resolution targets in complex backgrounds. Continuing to introduce the ODConv structure into the model, YOLO-OS is more advantageous in fishing boat detection accuracy, with AP values improved by 1.0% compared to YOLO-ES. This demonstrates that combining the ELAN-ODConv and ELANH-SPDC structures enhances the model’s performance in detecting small targets. YOLO-Vessel surpasses YOLOv7, YOLO-ES, and YOLO-OS in detecting fishing boats, highlighting the ASFF network’s effectiveness in improving detection performance for the IDetect head. In addition, YOLO-Vessel also demonstrates an advantage in detecting large-scale vessels such as bulk carriers and container ships. Its AP values for these categories are improved by 3.7% and 2.7%, respectively, compared to YOLOv7. This indicates that the combination of ELANH-SPDC structure, ELAN-ODConv structure, and ASFFPredict structure can fully learn more visual features of ships and thus improve the model’s performance of ship detection in adverse weather, especially detecting critical hull parts and reducing the number of false alarms. The final YOLO-Vessel has good performance for overall ship detection and can significantly improve the accuracy of ship detection under adverse weather conditions at sea.
2. In terms of inference speed, the YOLO-ES model achieves the fastest detection speed. However, with the introduction of the ELAN-ODConv module, the network’s inference speed slightly decreases, but the model’s accuracy improves. Therefore, the YOLO-Vessel model trades off accuracy and inference speed, compensating for the slight reduction in speed to enhance detection accuracy.
3. Regarding model computation, compared to the original model, YOLO-ES, YOLO-OS, and YOLO-Vessel reduced GFLOPS by 8.4, 10.7, and 2.4, respectively. The significant reduction in computational demands alleviates the computational burden on the machine. Furthermore, the YOLO-Vessel model incorporates adaptively spatial feature fusion and dynamic convolution techniques for ship detection, enhancing detection performance. It achieves the highest F1 score in ship image detection, surpassing the original model by 3%.

**Figure 10:**
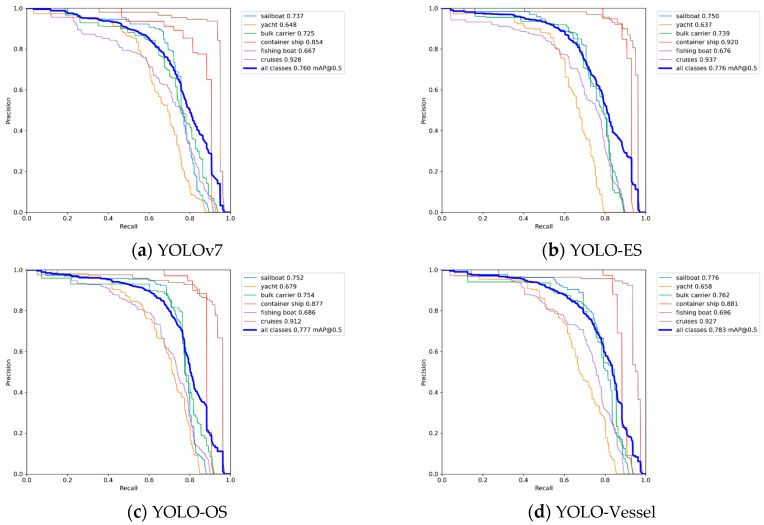
Precision-Recall curves for different improved models.

Figure 10 illustrates the Precision-Recall (P-R) curve. The area enclosed by the P-R curve and the coordinate axes in the image represent the mAP value. It can be observed that the mAP value of the YOLO-Vessel is higher than that of other improved models. The improved model shows a slight enhancement at different recall rates, indicating the effectiveness of the proposed improvements in enhancing the ship detection performance of the model.

**Figure 11:**
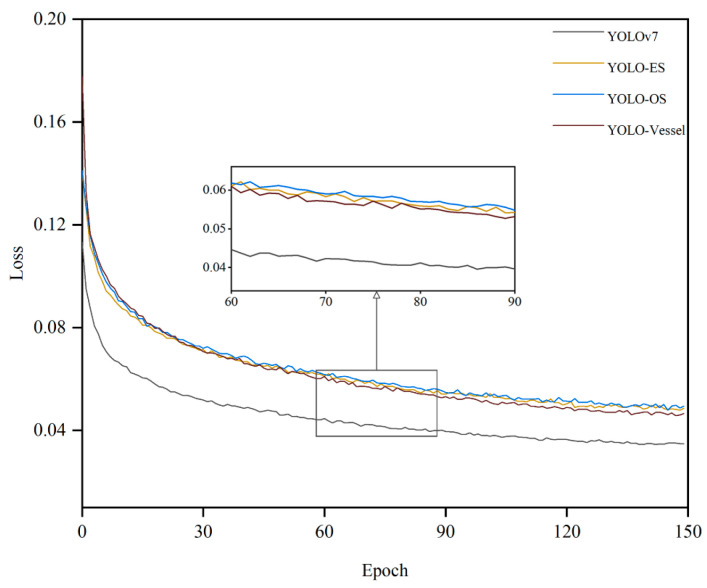
Comparison of loss values for different improved models of YOLOv7.

As Figure 11 demonstrates the comparison of the loss values of the original model with different improvements. Specifically, the loss curve of the YOLO-Vessel exhibits a swifter decline during initial training and converges more stably in the final phase compared to the other models, indicating that ELAN-ODConv and ELANH-SPDC can effectively extract features, resulting in a faster decrease in loss values and optimal model detection performance.

**Figure 12:**
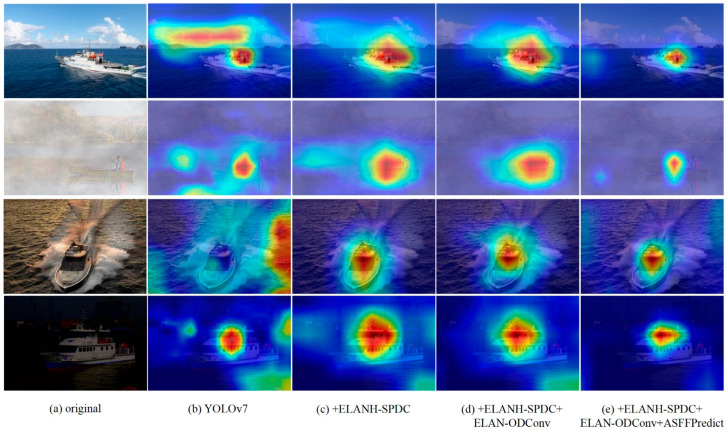
The heat map of the improved model of YOLOv7 introduces different modules for detecting ships.

We also validate the effectiveness of introducing SPDC, ODConv, and ASFF. We employ gradient-weighted class activation mapping (Grad-CAM) to visualize heatmaps for the four models, explaining the significance of feature capture in the improved models. In the Grad-CAM heatmaps, deeper colors indicate regions contributing more to ship detection in the heatmap. Figure 12a displays the original image of the target to be detected. As depicted in Figure 12b, the heatmaps of YOLOv7 exhibit poor ship detection performance under adverse weather conditions, with instances of heatmap region misalignment or sparsity. In Figure 12c, introducing the ELANH-SPDC module mitigates the impact of adverse weather or noise to some extent, capturing the approximate location of ships and reducing interference in learning ship features under low resolution. Figure 12d illustrates the continued improvement by introducing the ELAN-ODConv, enhancing the backbone network’s ability to extract ship features and reducing the number of irrelevant regions in the heatmap. Finally, in Figure 12e, introducing the ASFFPredict structure further refines the localization of ship targets, narrowing the heatmap range and indicating an increased focus on ship features.

**Figure 13:**
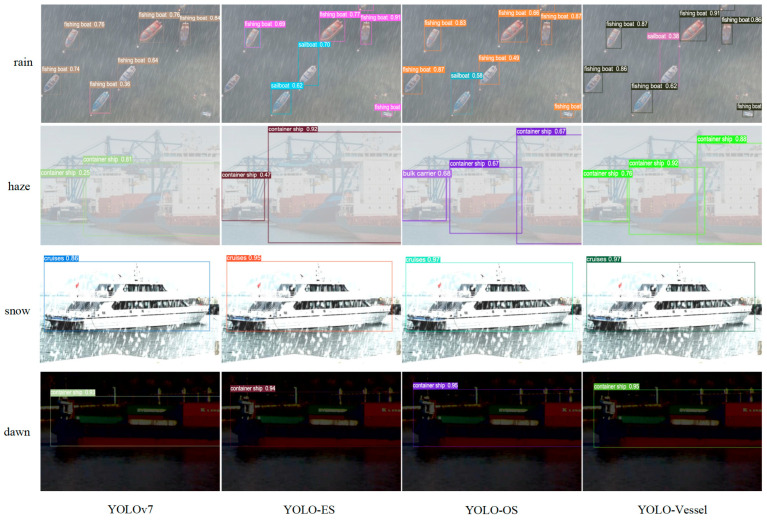
The experimental comparison of YOLO-Vessel with YOLOv7, YOLO-ES, and YOLO-OS models is presented in maritime vessel detection. From top to bottom, they represent vessel detection in rainy, hazy, snowy, and dawn weather conditions.

Figure 13 compares ship image detection performance under challenging conditions, including adverse weather, small target occlusion, and low lighting. This comparison involves YOLOv7, YOLO-ES, YOLO-OS, and YOLO-Vessel. As shown in Figure 13, YOLOv7 has problems with missed and false ship detection in all types of images. The same is true for YOLO-EO and YOLO-OA, and problems include, for example, insufficient accuracy in identifying frame positioning. However, YOLO-Vessel can successfully perform the identification task and locate the target vessel more accurately in the images.

In the rainy and hazy conditions shown in Figure 13, YOLOv7 has missed ships because of the small size of individual vessels and the less distinct hull outline, which is very close to the texture of the background. In addition, in the snow and dawn conditions shown in Figure 13, the detection performance remains poor because YOLOv7 does not locate the target size well, and hence the confidence level of detecting the ship targets is low. In contrast, YOLO-ES has good detection results and can detect multiple and small ships simultaneously, indicating the effectiveness of ELANH-SPDC, but it detects frame calibration imprecisely. In Figure 13, YOLO-OS and YOLO-Vessel can successfully identify the small target position, demonstrating the efficacy of ASFFPredict and ELANH-ODConv. In addition, YOLO-Vessel can identify more small target ship positions with smaller objects, proving that YOLO-Vessel is more robust in images of ships under extreme adverse weather conditions.

#### 4.5.4. Comparison with Other Algorithms

To further evaluate the detection performance of YOLO-Vessel, this paper compares the algorithms with several mainstream algorithms, employing the same synthetic ship dataset, and including Faster R-CNN, Fast R-CNN, Mask R-CNN, Cascade R-CNN, SSD, YOLOv3, YOLOv4, YOLOv5, YOLOv7, and YOLOv8. The experimental results in Table 5 demonstrate that the proposed YOLO-Vessel outperforms other mainstream algorithms. Specifically, the performance of Faster R-CNN, Fast R-CNN, Mask R-CNN, Cascade R-CNN, SSD, YOLOv3, and YOLOv4 is significantly worse than that of other algorithms, and it is usually challenging to perform ship detection robustly at sea level in adverse weather in marine ship detection applications. In comparison, the detection based on YOLOv5, YOLOv7, and YOLOv8 performs better. Regarding recall, while Faster R-CNN, YOLOv5m, and YOLOv5x show slightly better performance, YOLO-Vessel achieves the highest F1 score at 78%. In terms of model complexity, YOLO-Vessel has 100.3 GFLOPS, which is less than YOLOv5l, YOLOv5x, YOLOv7, YOLOv7x, YOLOv8l, and YOLOv8x, although greater than YOLOv8m at 78.7 MB. However, it achieves excellent results in mAP. Regarding inference time, YOLOv8n has the shortest inference time and the fastest speed but has insufficient detection accuracy. YOLO-Vessel has a trade-off between detection accuracy and inference speed, achieving an mAP of 78.3%, making it more advantageous than other algorithms. Furthermore, its accuracy in detecting sailboats, a crucial aspect of maritime surveillance, excelled, reaching a remarkable 77.6%. This indicates that YOLO-Vessel is also more accurate and robust for small ship detection in complex sea conditions. In conclusion, compared with other mainstream models, YOLO-Vessel has a higher accuracy advantage and is more suitable for ship detection in adverse weather.

**Figure 14:**
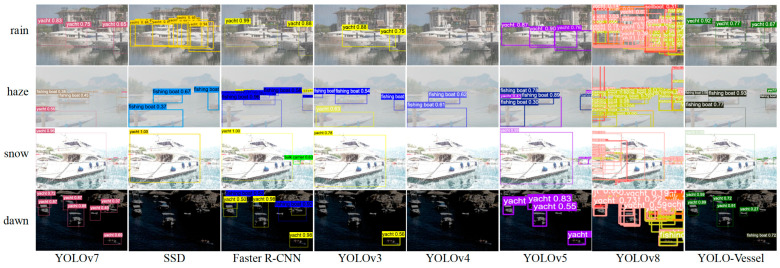
From top to bottom: representing the results of complex background ship detection at sea in rainy, hazy, snowy, and dawn weather, respectively.

**Figure 15:**
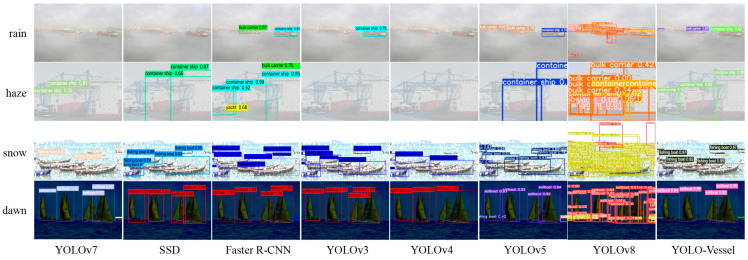
From top to bottom: the results of multi-objective ship detection at sea representing rainy, hazy, snowy, and dawn weather, respectively.

**Figure 16:**
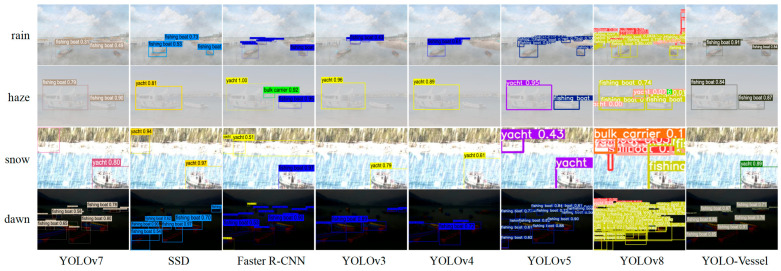
From top to bottom: small target ship detection results at sea representing rainy, hazy, snowy, and dawn weather, respectively.

Comparisons of ship detection performance in rainy, hazy, snowy, and dawn weather are shown in Figure 14, Figure 15 and Figure 16. The other models in the comparison can achieve accurate ship detection in adverse weather, but errors can occur in other complex sea conditions. Due to the sizeable similar ship outline background interference and small ship occlusion, YOLOv8 cannot capture the features of small ships well, and multiple misidentified detection frames appear, resulting in less accurate ship detection at sea. In the dawn environment, the SSD algorithm misses many ships. The Faster R-CNN algorithm has improved at detecting localization recognition, but the target confidence still needs to be higher. YOLOv4 misses ship detection in the snowy environment, while YOLOv3 and YOLOv5 have better feature extraction ability, but there is a false detection situation, which may mistakenly detect the background as a ship. In contrast, the YOLO-Vessel model improves the prediction network by using ASFF, enabling it to fuse targets’ structural features at different scales, detect more ships in complex sea conditions, and achieve better detection performance.

#### 4.5.5. Experiments on Realistic Ship Detection

**Figure 17:**
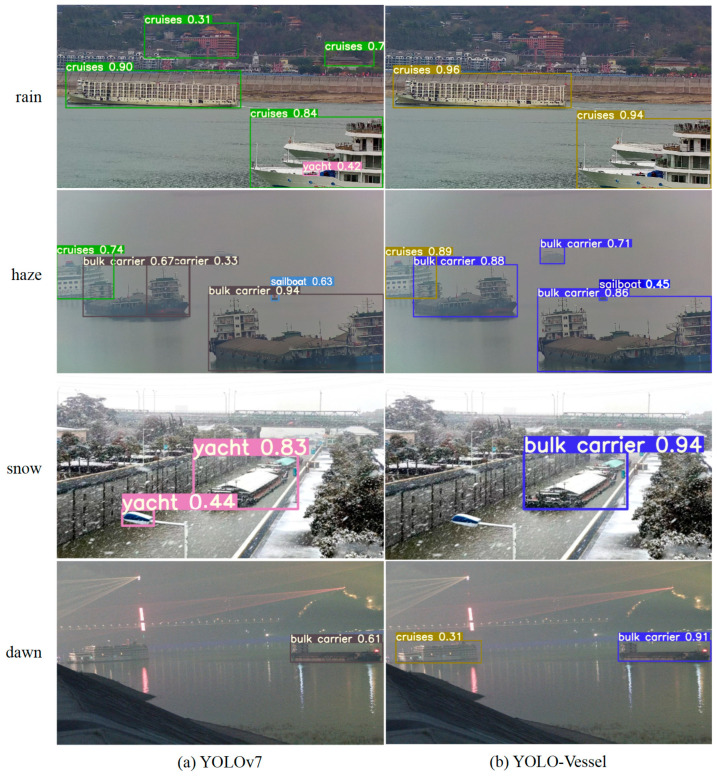
Ship detection experiments in authentic adverse weather images. From left to right: ship detection results for (**a**) YOLOv7 and (**b**) YOLO-Vessel in rainy images, haze, snow, and dawn weather, respectively.

This section further demonstrates the YOLO-Vessel model’s detection performance in a realistic environment, as illustrated in Figure 17.

Compared with the YOLOv7 algorithm, YOLO-Vessel can extract different types of ship features under different adverse weather conditions and perform target detection for six common types of ships with good recognition results. From the ship detection results in rainy, hazy, snowy, and dawn weather, we can conclude the following:

1. As shown in the rain of Figure 17, YOLOv7 mistakenly identifies the house building background as a ship in a rainy environment; in the same case, as shown in the snow in Figure 17, YOLOv7 incorrectly detects street light as some other vessels in snowy weather, while YOLO-Vessel can correctly detect the ship category and location with 94% confidence, which solves the complex background interference problem.
2. As shown in the haze of Figure 17, under hazy weather conditions, YOLOv7 successfully detected large bulk carriers and cruise ships near the shore but failed to detect small target vessels in the distance. In contrast, YOLO-Vessel significantly improved recognition of small ships in haze weather, effectively reducing the probability of missed detections.
3. As shown in the dawn of Figure 17, in the dawn environment, YOLOv7 only detects one ship, and the YOLO-Vessel model avoids missed detection and detects all the cruise ships and bulk carrier in the picture.

In conclusion, YOLO-Vessel outperforms YOLOv7 on the real adverse weather dataset, demonstrating its superiority in challenging conditions and further validating the effectiveness of YOLO-Vessel for ship detection in complex backgrounds.

## 5. Conclusions

In addressing the challenges posed by adverse weather, complex background interference in ship detection images, and multiscale ship target detection, this paper introduces ODConv to optimize the YOLOv7 model. It fully uses four-dimensional weights to learn image features, achieving efficient feature extraction and addressing the challenges of missed and false detections in complex backgrounds. Moreover, the model’s detection accuracy is enhanced by introducing the ELANH-SPDC module, guided by SPDC, in the head network to preserve finer feature details. Furthermore, incorporating ASFFPredict into the detection head allows for aggregating more semantic information, enabling more comprehensive feature fusion. This effectively tackles the issues related to detecting ships at various scales, addressing the uneven distribution of target features and enhancing the detection performance for smaller targets. Under the sample constraint, this paper improves the robustness of YOLO-Vessel for target detection in adverse weather using a mixed weather ship image training mechanism. Compared with other YOLO series models, the YOLO-Vessel method trades off complexity and detection accuracy. It can detect many maritime vessels in real-time with high accuracy. In general, the mAP of YOLO-Vessel reaches 78.3%, the detection speed is 8.0 ms/frame, and the GFLOPS is 100.8, thus satisfying real-time ship detection at sea. In our future research, we plan to explore lightweight techniques for the primary backbone of the model to reduce parameter count and model size. This may include methodologies such as model pruning, quantization, and utilizing lightweight convolution modules. We aim to develop models better suited for deployment on mobile devices, enhancing their applicability to maritime ship detection tasks.

## Figures and Tables

**Table 1:** Detection results of SPDC applied at different positions in the YOLOv7 head network.

Model	Position	mAP (%)	F1 (%)	GFLOPS	R (%)	Infer (ms)
YOLOv7 (baseline)	-	76.0	75	103.2	69.5	9.0
YOLOv7-SPDC-1	p_out3	77.4	76	97.7	72.2	6.9
YOLOv7-SPDC-2	p_out4	75.8	76	97.7	68.7	6.6
YOLOv7-SPDC-3	p_out5	77.6	76	94.8	70.9	7.0

**Table 2:** Detection results of replacing ordinary convolutions with ODConv at different positions in the ELAN module.

Model	Position	mAP(%)	F1(%)	GFLOPS	R(%)	Infer(ms)
YOLOv7 (baseline)	-	76.0	75	103.2	69.5	9.0
YOLOv7-ODConv-a	a	77.1	76	100.9	73.5	9.5
YOLOv7-ODConv-b	b	75.9	75	100.9	70.0	8.1
YOLOv7-ODConv-c	c	77.6	77	100.9	69.7	9.3
YOLOv7-ODConv-d	d	74.7	74	99.3	69.5	8.5

**Table 3:** YOLOv7 models with different improvements.

Model	SPPFCSPC	ELANH-SPDC	ELAN-ODConv	ASFFPredict
YOLO-ES	√	√	×	×
YOLO-OS	√	√	√	×
YOLO-Vessel	√	√	√	√

**Table 4:** Performance comparison of the original model with different improved models.

Model	AP (%)						mAP (%)	F1 (%)	GFLOPS	Infer (ms)
	Sailboat	Yacht	Bulk Carrier	Container Ship	Fishing Boat	Cruises				
YOLOv7	73.7	64.8	72.5	85.4	66.7	92.8	76	75	103.2	9.0
YOLO-ES	75.0	63.7	73.9	92.0	67.6	93.7	77.6	76	94.8	7.0
YOLO-OS	75.2	67.9	75.4	87.7	68.6	91.2	77.7	77	92.5	7.8
YOLO-Vessel	77.6	65.8	76.2	88.1	69.6	92.7	78.3	78	100.8	8.0

**Table 5:** Comparison of experimental results.

Model	AP (%)						mAP (%)	F1 (%)	GFLOPS	R	Infer (ms)
	Sailboat	Yacht	Bulk Carrier	Container Ship	Fishing Boat	Cruises					
Faster R-CNN	66.2	53.1	65.8	80.0	51.2	83.9	66.7	55	-	71.7	16.9
Fast R-CNN	49.7	47.8	57.4	56.8	30.4	88.6	55.1	-	-	-	-
Mask R-CNN	57.0	50.5	62.5	80.5	39.1	89.9	63.3	-	-	-	-
Cascade R-CNN	57.1	55.2	70.5	78.3	40.3	90.9	65.4	-	-	-	-
SSD	59.7	52.5	65.6	84.8	50.5	89.8	67.1	69	-	57.9	8.1
YOLOv3	72.2	55.6	64.6	85.4	51.6	93.7	70.5	68	-	56.6	10.6
YOLOv4	64.3	45.4	32.6	48.8	51.2	78.7	53.5	45	-	35.3	12.7
YOLOv5s	71.2	61.7	64.0	82.0	60.7	91.6	71.9	71	16.3	67.2	10.8
YOLOv5m	72.0	64.3	73.3	86.8	61.1	91.6	74.9	75	50.3	72.8	11.6
YOLOv5l	71.9	64.3	70.6	87.0	65.5	91.8	75.2	75	114.0	68.7	13.0
YOLOv5x	73.4	59.1	71.8	87.4	64.0	91.9	74.6	75	217.0	70.8	16.3
YOLOv7-tiny	70.0	57.1	60.9	83.2	56.4	89.7	69.5	68	13.1	65.8	7.9
YOLOv7	73.7	64.8	72.5	85.4	66.7	92.8	76.0	75	103.2	69.5	9.0
YOLOv7x	73.8	66.8	76.7	88.0	67.4	93.0	77.6	75	188.1	69.9	13.0
YOLOv8n	71.1	53.3	66.3	84.9	57.6	91.5	70.8	70	8.1	63.5	6.6
YOLOv8s	73.6	60.7	63.8	84.3	58.2	90.7	71.9	70	28.4	65.2	8.8
YOLOv8m	75.5	56.2	68.8	85.7	60.7	90.3	72.9	71	78.7	66.1	9.8
YOLOv8l	74.3	57.2	68.5	81.7	59.8	90.3	72.0	70	164.8	65.5	12.7
YOLOv8x	74.1	59.2	69.0	85.7	64.5	91.2	74.0	72	257.4	66.6	15.5
YOLO-Vessel	77.6	65.8	76.2	88.1	69.6	92.7	78.3	78	100.8	70.7	8.4

## Data Availability

The data presented in this study are available on request from the corresponding author.
